# Immunization with MVA-based vaccines protects K18-hACE2 mice from SARS-CoV-2 infection-associated inflammatory lesions in brains

**DOI:** 10.3389/fimmu.2026.1788665

**Published:** 2026-03-10

**Authors:** Małgorzata Rosiak, Sabrina Clever, Eva Leitzen, Georg Beythien, Lukas Mathias Michaely, Sandra Lockow, Lisa Allnoch, Malgorzata Ciurkiewicz, Christian Meyer zu Natrup, Tamara Tuchel, Alina Tscherne, Leonard Limpinsel, Gerd Sutter, Asisa Volz, Wolfgang Baumgärtner, Kirsten Hülskötter, Katharina Manuela Gregor

**Affiliations:** 1 Department of Pathology, University of Veterinary Medicine Hannover, Hannover, Germany; 2 Center for Systems Neuroscience, Hannover, Germany; 3 Institute of Virology and Research Center for Emerging Infections and Zoonoses, University of Veterinary Medicine Hannover, Hannover, Germany; 4 Division of Virology, Department of Veterinary Sciences, LMU Munich, Munich, Germany

**Keywords:** COVID-19, K18-hACE2 mice, modified vaccinia ankara-based vaccines, SARS-CoV-2, vaccines

## Abstract

Severe acute respiratory syndrome coronavirus 2 (SARS-CoV-2) is known as the etiological agent of coronavirus disease 2019 (COVID-19). Extrapulmonary manifestations of COVID-19 have gained increasing recognition as significant contributors to disease severity and long-term complications. The aim of this study is to investigate the neuroprotective properties of vaccines based on modified Vaccinia Virus Ankara (MVA) against SARS-CoV-2 infection in K18-hACE2 mice using different immunization protocols. Animals received PBS, vector, recombinant MVA expressing native (S) or stabilized (ST) SARS-CoV-2 spike protein, nucleocapsid protein (N) or both ST and N protein twice, followed by infection with SARS-CoV-2 four weeks later. In further experiments, mice were immunized only once and infected two days (Emergency experiment) or four weeks (Prime experiment) later. Both the control groups and the animals immunized with vaccines expressing only N-protein showed mild to moderate, lymphohistiocytic meningoencephalitis, microgliosis and numerous virus antigen-positive neurons in the brains and to a lesser extent in the retinas. Groups immunized four weeks prior to infection with vaccines containing viral spike protein showed no or minimal inflammatory changes and no neuroinvasion. Animals infected two days after immunization showed milder lesions than unvaccinated control groups.

## 1 Introduction

Severe acute respiratory syndrome coronavirus 2 (SARS-CoV-2), the etiological agent of coronavirus disease 2019 (COVID-19) has caused over 7 million of deaths worldwide till December 2025 (1).

Considering the potential for SARS-CoV-2 reinfection, even in individuals who have fully recovered from COVID-19, and the limited therapeutic strategies available, vaccination appears to be the most effective measure to prevent severe disease progression and associated complications, although variable breakthrough infection rates have been reported (2–4). Neurological manifestations including headache, dizziness, myalgia/fatigue, anorexia, anosmia and ageusia are quite frequently observed in COVID-19 patients with reported prevalence ranging between 36,4% and 100% (5–8). Although less common, the ocular system involvement including conjunctival congestion, conjunctivitis, and retinal changes has been reported as well (9). In addition, there is circumstantial evidence for the presence of SARS-CoV-2 in the brain of infected individuals (10–13). Therefore, assessing the neuroprotective capacity of vaccines is crucial and could help predicting whether immunized individuals exposed to the infection remain at risk of developing neurological complications.

K18-hACE2 transgenic mice, in which the cytokeratin-18 (K18) promoter drives expression of human angiotensin-converting enzyme 2 (hACE2) (14), represent a well-established model for severe SARS-CoV-2 infection with widespread neuroinvasion and mild to moderate, lymphohistiocytic, perivascular meningoencephalitis (15–18). Robust central nervous system (CNS) involvement in this animal model provides an opportunity for testing the neuroprotective features of vaccines and therapeutics.

Modified Vaccinia Virus Ankara (MVA) is a highly attenuated vaccinia virus licensed as a vaccine against smallpox and Mpox and it is also used as a vector system for the development of new candidate vaccines against a variety of pathogens (19). MVA-based vaccine candidates containing native (20–27) or modified (21, 27–29) spike proteins (S) or both S and nucleocapsid proteins (N) (20, 21) of SARS-CoV-2 were tested in K18-hACE2 mice (22–24, 27–29), BALB/cJ mice (21, 26), C57BL/6J mice (21, 22), Syrian hamsters (20, 27), rhesus macaques (21) and African green monkeys (20). However, most of these studies focus on the pathology of the respiratory tract rather than the CNS.

This study utilizes histology and immunohistochemistry (IHC) to characterize the inflammatory response and distribution of viral antigen expression in the brain of vaccinated and SARS-CoV-2-infected K18-hACE2 mice with special emphasis on the infection or its lack in eyes, in addition to previously published data on clinical manifestation, immune responses (neutralizing antibodies, N-specific antibodies, S- and N-specific T cells), as well as viral load in the lung and brain based on molecular analyses (27, 30).

## 2 Methods

### 2.1 Animal experiments and study design

All experiments were performed in accordance with the European Directive 2010/63/EU and the national Animal Welfare Act with the approval of the Lower Saxony State Office for Consumer Protection and Food Safety (LAVES, Lower Saxony, Germany; file number 33.8-42502-04-20/3440) in BSL-3 facilities at the Research Center for Emerging Infections and Zoonoses (RIZ), University of Veterinary Medicine Hanover Germany. Female and male K18-hACE2 mice (B6.Cg-Tg (K18-ACE2)2Prlmn/J) were immunized by intramuscular injection into the quadriceps muscle twice (day 0 and 21) for the Booster experiment or once for the Prime (day 0) and Emergency (day 0) studies. In each experiment all groups were infected intranasally with SARS-CoV-2 (3.6 × 10*^4^* TCID50, Germany/BavPat1/2020 strain, NR-52370). In order to keep the numbers of animals at the minimum needed for significant interpretation of the data, they were allocated to experimental groups as they were provided, and no randomization was applied. Comparable numbers of male and female mice were included whenever possible, although this could not always be achieved. The intranasal infection took place under general anesthesia through intraperitoneal administration of midazolam/medetomidine (5 mg/0.5 mg/kg respectively). Antagonization was carried out by subcutaneous injection of flumazenil/atipamezole (0.5 mg/2.5 mg/kg respectively).

**Figure 1:**
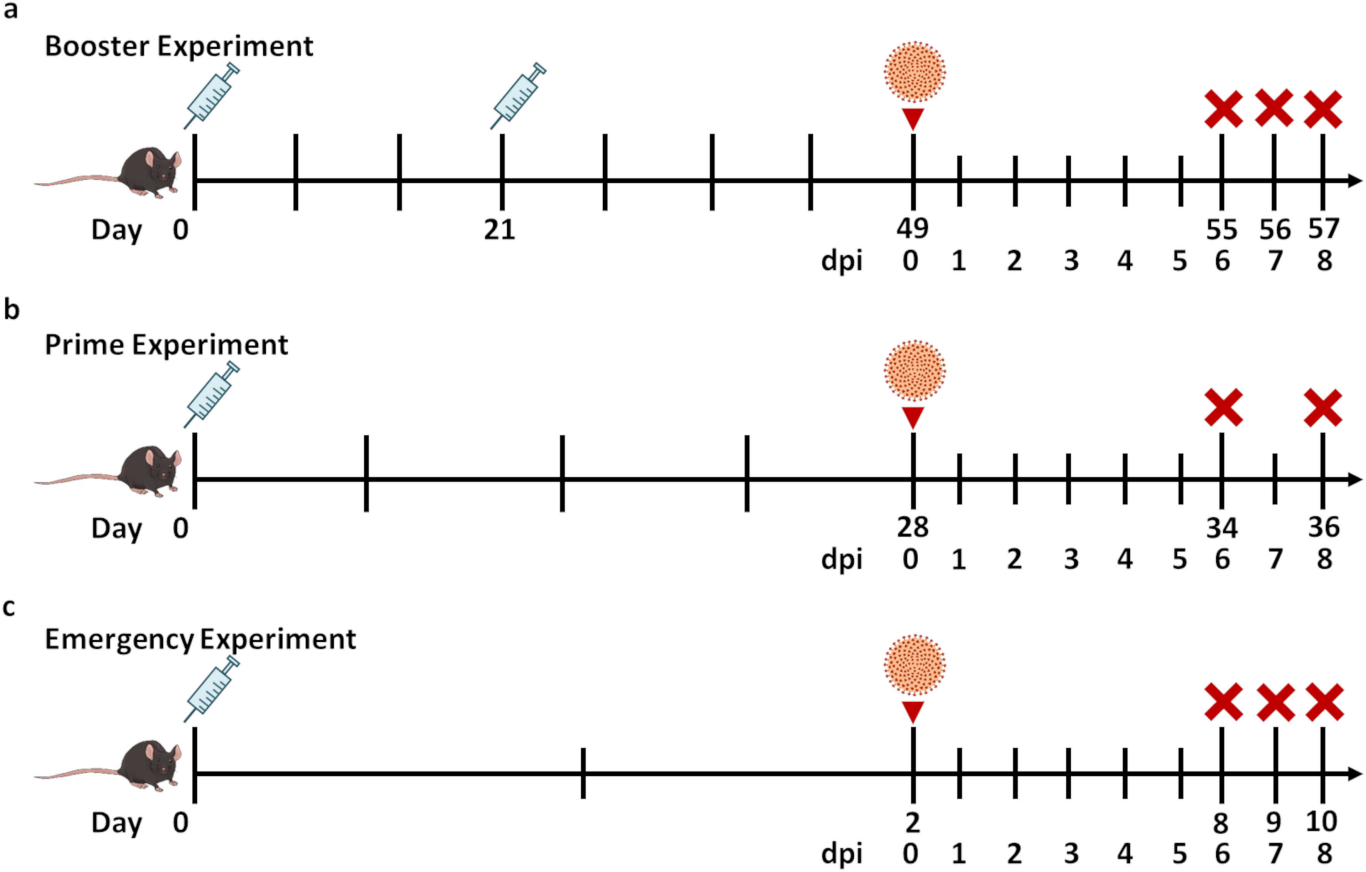
Study design. Male and female K18-hACE2 mice received PBS, Modified Vaccinia Virus Ankara wild type (MVA-WT), recombinant MVA expressing native (S) or stabilized (ST) SARS-CoV-2 spike protein, nucleocapsid protein (N) or both ST and N proteins prior to intranasal SARS-CoV-2 infection (3.6 × 10^4^ TCID50, Germany/BavPat1/2020 strain, NR-52370). **(a)** Booster experiment - mice were vaccinated twice (day 0 and 21) and infected four weeks later. **(b)** Prime experiment - mice were immunized only once and infected four weeks later. **(c)** Emergency experiment – animals were vaccinated once and infected two days later. Mice were sacrificed 6-, 7- and 8 days post infection (dpi). The syringe icon indicates vaccination, the virus icon denotes SARS-CoV-2 infection in the animals, and the red cross marks the day of euthanasia.

In the Booster experiment (**Figure 1a**) animals underwent a two-dose vaccination schedule (day 0 and 21) including either PBS (*n* = 4), MVA wild type (MVA-WT) as a vector control (*n* = 4), recombinant MVA expressing native (MVA-SARS-2-S; *n* = 3) or stabilized (MVA-SARS-2-ST; *n* = 4)) SARS-CoV-2 spike protein, nucleocapsid protein (MVA-SARS-2-N; *n* = 5) or both ST and N proteins (MVA-SARS-2-ST/N; *n* = 8) and were infected four weeks after the last immunization. Mice from the PBS, MVA-WT and MVA-SARS-2-N groups reached humane end point and were sacrificed at 6 dpi (with exception of two animals from MVA-SARS-2-N – 7 dpi). Animals from other groups survived until the end of experiment and were sacrificed at 8 dpi.

In the Prime study (**Figure 1b**) mice received once (day 0) either PBS (*n* = 4), MVA-WT (*n* = 4), MVA-SARS-2-ST (*n* = 8), MVA-SARS-2-N (*n* = 8) or MVA-SARS-2-ST/N (*n* = 8) and were infected four weeks after the last vaccination. In the Prime experiment all mice from the PBS, MVA-WT and MVA-SARS-2-N groups reached human endpoint and were sacrificed at 6 dpi. All the other groups were sacrificed as planned at 8 dpi except for one animal from MVA-SARS-2-ST group that had to be sacrificed at 6 dpi.

The Emergency experiment (**Figure 1c**) included mice that were immunized once with either PBS (*n* = 7), MVA-WT (*n* = 7) or MVA-SARS-2-ST/N (*n* = 6) and infected two days after the vaccination. Animals were sacrificed at 6, 7 and 8 dpi. In this experiment, all animals from the PBS and MVA-WT group (at dpi 6), one animal from the MVA-SARS-2-ST/N group (at 6 dpi) as well as 2 additional animals from the MVA-SARS-2-ST/N group (at dpi 7) reached the humane end point and were sacrificed. The other three animals immunized with the MVA-SARS-2-ST/N vaccination survived until the end of the experiment at 8 dpi.

In all experiments euthanasia was performed through intraperitoneal injection of ketamine/xylazine (100/5.0 mg/kg respectively) to induce unconsciousness, followed by exsanguination via cardiac puncture.

Parts of these animal experiments, including details on animal husbandry and clinical data, have been published previously (27, 30).

### 2.2 Histology and immunohistochemistry

Tissue samples were fixed for 72 hours (brains) or 3 weeks (eyes) in 10% neutral buffered formalin. The difference in duration of the fixation was related to the different inactivation time for the virus regarding soft (brain) and hard (eyes within the skull) tissues, which delayed the tissue processing of the eyes. Subsequently the samples were embedded in paraffin wax and cut into 2-4 μm thick sagittal serial sections for histological and immunohistochemical analysis. For histological evaluation, sections were routinely stained with hematoxylin and eosin (HE). The avidin-biotin-peroxidase complex method was implemented with the primary antibodies targeting anti SARS-CoV-2-NP (SARS-CoV-2 nucleocapsid protein, 40143-MM05, Sino Biological, Beijing, China) anti Iba-1 (ionized calcium-binding adapter molecule 1; #019-197, FUJIFILM Wako Pure Chemical Corporation, Richmond, USA; 1:8000) and anti CD3 (#A0452, Dako Agilent, Santa Clara, USA; 1:200) for SARS-CoV-2-NP, microglia/macrophages and T cells, respectively, as previously described (31). As negative controls, primary antibodies were replaced either by normal rabbit serum for Iba-1 and CD3 (1:3000; #R4505, Sigma-Aldrich Chemie GmbH, Tauffkirchen, Germany) or by ascites fluid from non-immunized BALB/c mice in case of SARS-CoV-2-NP staining (1:1000; #BL CL8100, Cedarlane^®^, biologo, Kronshagen, Germany).

The scoring was performed by a veterinary pathologist in a blinded manner. For evaluation of brain lesions, longitudinal brain sections were divided into two areas (cerebrum with brain stem and cerebellum), which were assessed separately using a previously published semi-quantitative scoring system for the HE stained slides (31) and for SARS-CoV-2-NP staining as follows: 0 = no positive neurons, 1 = focal to multifocal areas with positive neurons (less than 30% of the evaluated area), 2 = multifocal to coalescing areas with positive neurons (between 30% and 70% of the evaluated area), 3 = coalescing to diffuse area with positive neurons (more than 70% of the evaluated area). Globes, including retinal layers, were examined separately and scored using a dichotomous scoring system (0 = no; 1 = yes) for both histological lesions and SARS-CoV-2-NP immunoreactivity.

### 2.3 Digital image analysis

Tissue sections were digitalized using the Olympus VS200 slide scanner (40x magnification, Olympus Deutschland GmbH, Hamburg, Germany). Digital quantification of the immunohistochemical staining was performed using the open source software Qupath (version 0.4.3) (32) as previously described (31).

### 2.4 Statistical analysis

To investigate significant differences between the groups, data were analyzed using SPSS for Windows™ v29 (IBM^®^ SPSS^®^ Statistics, SPSS Inc., Chicago, IL, USA). Specifically, the Kruskal-Wallis test followed by the Dunn-Bonferroni procedure were used. Statistical significance was accepted at p-value of ≤ 0.05 (*). The graphs were created with GraphPad Prism version 10.2.3 (GraphPad Software, Inc., San Diego, CA, USA) for Windows™ software.

## 3 Results

The data regarding the development of the vaccine, clinical condition of the animals, viral loads in the brain and quantification of the immunohistochemistry for the virus were previously published (27, 30).

As depicted in **Figure 1**, male and female K18-hACE2 mice received PBS, MVA-WT, MVA-SARS-2-S, MVA-SARS-2-ST, MVA-SARS-2-N or MVA-SARS-2-ST/N prior to intranasal SARS-CoV-2 infection (3.6 × 10^4^ TCID50, Germany/BavPat1/2020 strain, NR-52370). Mice were vaccinated either twice (day 0 and 21; Booster experiment) or once (day 0; Prime experiment) and infected four weeks later. In the Emergency experiment animals were vaccinated once (day 0) and infected two days later. Mice were sacrificed at 6-, 7- and 8 dpi.

### 3.1 Booster experiment

#### 3.1.1 Brains

**Figure 2:**
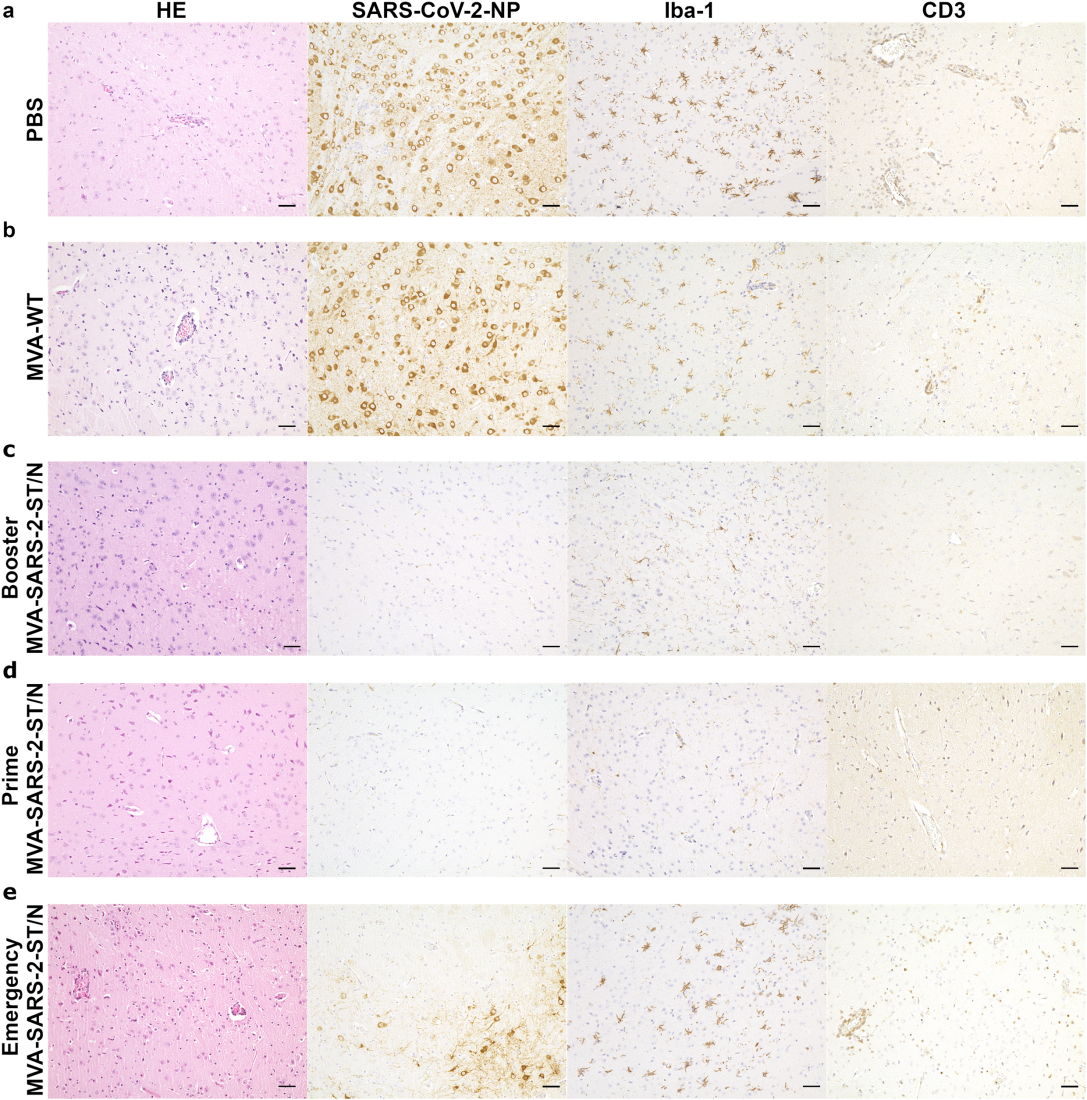
In contrast to infected animals from both the PBS [**(a)** 6 days post infection (dpi)] and MVA-WT [**(b)** 6 dpi] groups, which showed lymphohistiocytic meningoencephalitis with microgliosis and neuroinvasion, K18-hACE2 mice, vaccinated twice [Booster experiment, **(c)** 8 dpi] or once [Prime experiment, **(d)** 8 dpi] four weeks prior to infection with MVA-based vaccines expressing stabilized spike and nucleocapsid proteins (MVA-SARS-2-ST/N) showed no inflammatory lesions and no viral spread. Animals immunized with MVA-SARS-2-ST/N two days prior to infection [Emergency experiment, **(e)** 6 dpi (two left pictures) and at 8 dpi (two right pictures)] also developed lesions, but the inflammatory alterations and the immunopositivity for the viral antigen were less pronounced than in the controls. **(a, b)** Control animals showed lymphohistiocytic meningoencephalitis, numerous SARS-CoV-2-NP-positive neurons, spiky microglia, and predominantly perivascular T cell infiltration. **(c, d)** Animals vaccinated twice **(c)** or once **(d)** four weeks prior to infection with MVA-SARS-2-ST/N presented no inflammatory changes or neuroinvasion. **(e)** Animals immunized (MVA-SARS-2-ST/N) once two days prior to infection presented less severe inflammatory changes and a less pronounced spread of viral antigen than the control groups. Bars = 50µm.

**Figure 3:**
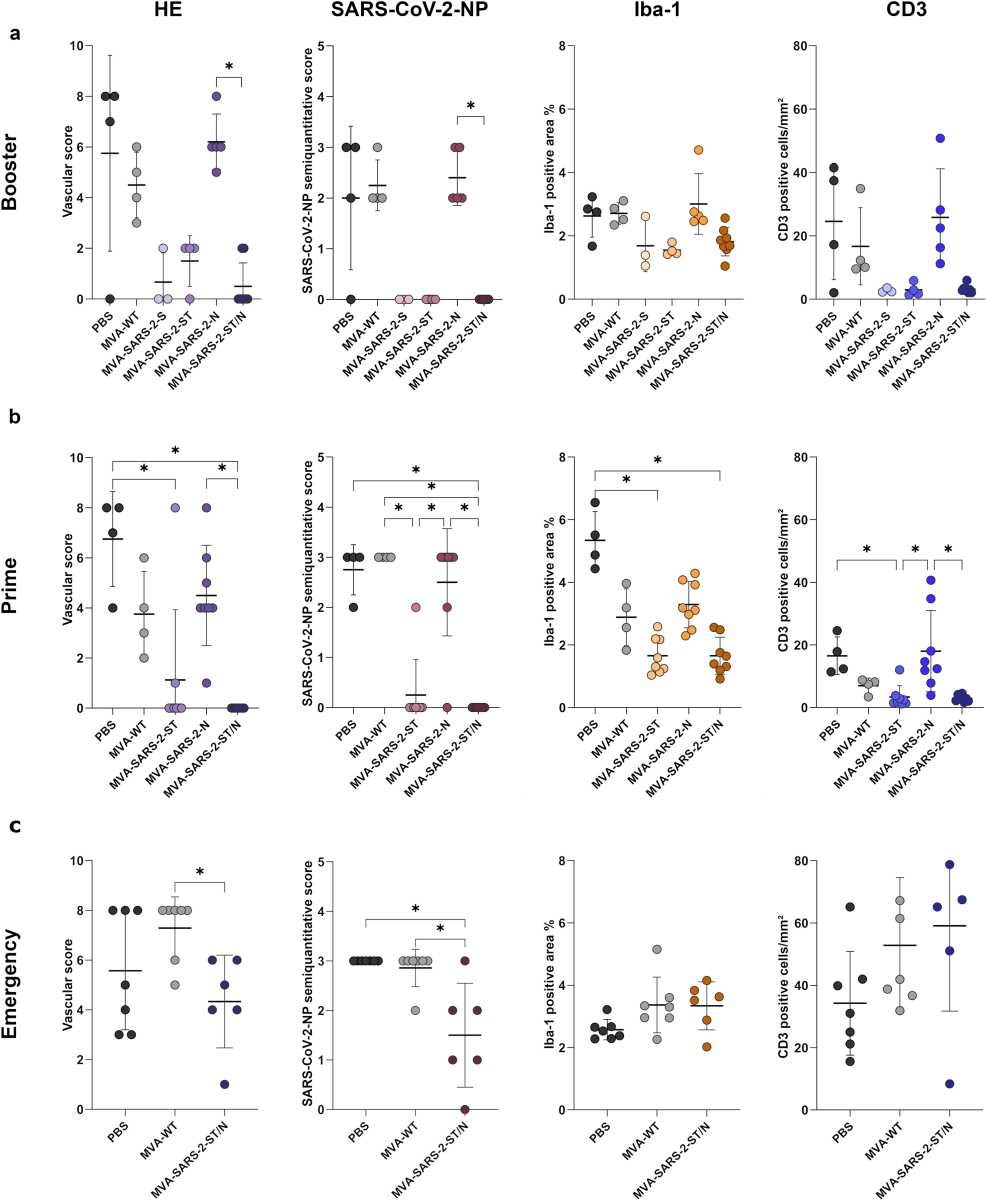
K18-hACE2 mice vaccinated once or twice four weeks prior to infection with MVA-based vaccines expressing native (S) or stabilized spike (ST) protein showed minimal or no inflammatory alterations, no presence of viral antigen or change in microglial morphology, and only minimal T cell infiltration in contrast to non-vaccinated animals from the PBS and MVA-WT groups and animals immunized with a MVA vaccine expressing the viral nucleocapsid (N) protein. Animals immunized with MVA-SARS-2-ST/N two days prior to infection also developed lesions, but the inflammatory changes and immunopositivity for viral antigen were less pronounced than in the control animals. **(a-c)** Semi-quantitative analysis of hematoxylin and eosin-stained and SARS-CoV-2-NP-immunostained slides as well as quantitative analysis of Iba-1-positive area and CD3-positive T cells in the cerebrum and brain stem of animals immunized with the Booster **(a)**, Prime **(b)** and Emergency **(c)** protocols. Data were tested using the Kruskal–Wallis test followed by Dunn–Bonferroni *post hoc* testing. Statistical significance was accepted at a p-value of ≤0.05 (*). The graphs show mean (solid line), individual values (dots), and standard deviation (vertical bars).

SARS-CoV-2 infected animals from control groups that received PBS (*n* = 4) or MVA-WT, (*n* = 4) as well as mice immunized with MVA-SARS-2-N (*n* = 5) showed similar histological and immunohistochemical features present in the cerebrum and brain stem, but not in the cerebellum. Changes included a mild to moderate, lymphohistiocytic meningoencephalitis, a multifocal to coalescing or diffuse distribution of SARS-CoV-2-NP-positive neurons, a multifocal to diffuse change of the resting microglial morphology to “spiky”, characterized by shorter, thicker processes and larger somas, and predominantly perivascular infiltration of T cells (**Figures 2a, b**). One control animal from the PBS group showed neither inflammatory changes nor the presence of SARS-CoV-2-positive neurons, spiky microglia or perivascular presence of T cells (*n* = 1/4). In contrast, animals immunized with MVA-SARS-2-S, MVA-SARS-2-ST and MVA-SARS-2-ST/N showed no or minimal histological changes, no SARS-CoV-2-NP-positive neurons, a diffuse morphology of resting microglia with long, thin processes and only occasional single T cells in the perivascular space and/or parenchyma (**Figure 2c**). In addition, these groups had a lower vascular score, lower Iba-1-positive area % and a reduced number of CD3-positive cells/mm^2^ compared to controls and MVA-SARS-2-N groups (**Figure 3a**). However, with the exception of the significantly lower vascular score and the semi-quantitative SARS-CoV-2-NP score of the MVA-SARS-2-ST/N compared to MVA-SARS-2-N group (p = 0.012 and 0.013, respectively), the differences did not reach statistical significance.

#### 3.1.2 Eyes

**Figure 4:**
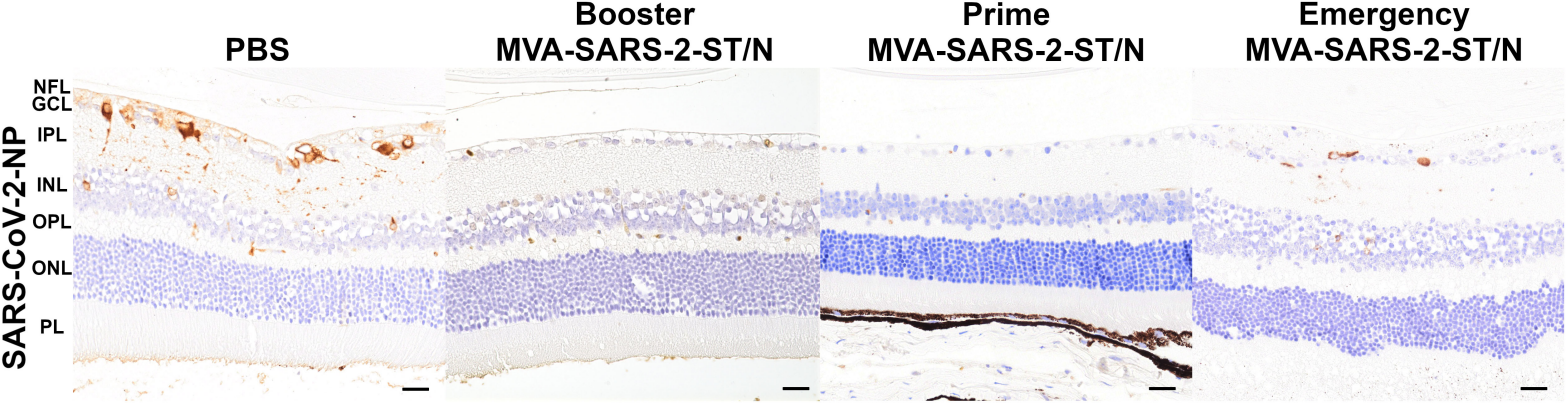
In contrast to the animals from control groups (PBS, 6 dpi), which showed multiple neurons (predominantly in the ganglion cell layer) positive for SARS-CoV-2 nucleocapsid protein (NP) in the retina, mice vaccinated four weeks prior to the infection with MVA-SARS-2-ST/N (Booster experiment and Prime experiment, 8 dpi) did not show any viral antigen immunopositivity. Only one animal immunized with MVA-SARS-2-ST/N two days prior to the infection displayed SARS-CoV-2-NP positive neurons in the retina (Emergency experiment, 7 dpi). Abbreviations indicate the retinal layers: NFL, nerve fiber layer; GCL, ganglion cell layer; IPL, inner plexiform layer; INL, inner nuclear layer; OPL, outer plexiform layer; ONL, outer nuclear layer; PL, photoreceptor layer; Bars = 50µm.

There were no morphological lesions present in the globes in any of the mice. Immunohistochemistry for SARS-CoV-2-NP revealed positive neurons in the retinas of mice that received PBS (*n* = 3/4) and MVA-SARS-2-N (*n* = 3/5). Most of the positive neurons were found in the ganglion cell layer, although in two animals from the PBS and one animal from the MVA-SARS-2-N groups few neurons in the inner nuclear layer showed SARS-CoV-2-NP immunopositivity as well. No positive cells were observed in any of the animals from the MVA-WT (*n* = 4), as well as the MVA-SARS-2-S (*n* = 3), MVA-SARS-2-ST (*n* = 4) and MVA-SARS-2-ST/N (n = 8) groups (**Figure 4**).

### 3.2 Prime experiment

#### 3.2.1 Brains

Infected animals that received PBS, MVA-WT or MVA-SARS-2-N presented similar histological and immunological features, such as mild to moderate, lymphohistiocytic meningoencephalitis, multifocal to coalescing or diffuse distribution of SARS-CoV-2-NP-positive neurons, multifocal to coalescing presence of spiky microglia with shorter, thicker processes and predominantly perivascular T cell infiltration (**Figures 2a, b**). One animal from the MVA-SARS-2-N group was devoid of these changes (*n* = 1/8). In contrast, mice vaccinated with MVA-SARS-2-ST and MVA-SARS-2-ST/N showed no or only minimal histological changes with lack of SARS-CoV-2-NP-positive neurons, predominantly resting microglia with long, thin processes, and only single T cells in the perivascular space and parenchyma (**Figure 2d**). One animal vaccinated with MVA-SARS-2-ST presented multifocal areas with SARS-CoV-2-NP-positive neurons along with inflammatory changes and microgliosis (*n* = 1/8). In addition, two animals from MVA-SARS-2-ST group showed focal to multifocal areas of microglia with spiky morphology in the absence of other inflammatory changes or viral antigen (*n* = 2/8).

Semiquantitative evaluation of the HE stained sections revealed a significantly lower vascular score in the MVA-SARS-2-ST and MVA-SARS-2-ST/N groups when compared to the PBS group (p = 0.030 and 0.002, respectively). Moreover, the vascular score of the MVA-SARS-2-ST/N group was significantly lower than that of animals immunized with MVA-SARS-2-N (p = 0.006). Semi-quantitative SARS-CoV-2-NP scores were significantly lower in the MVA-SARS-2-ST/N group than in the PBS (p = 0.027), MVA-WT (p = 0.007) and MVA-SARS-2-N (p = 0.007) groups. The SARS-CoV-2-NP scores were also significantly lower in the MVA-SARS-2-ST when compared to the MVA-WT (p = 0.016) and MVA-SARS-2-N (p = 0.019) groups. Quantification of Iba-1 staining showed significantly smaller Iba-1-positive area % in the MVA-SARS-2-ST and MVA-SARS-2-ST/N when compared to the PBS group (p = 0.002 and 0.003, respectively). The number of T cells in the cerebrum and brain stem was significantly lower in the MVA-SARS-2-ST group than in the PBS group (p = 0.019). In addition, animals immunized with MVA-SARS-2-N showed significantly higher number of T cells than MVA-SARS-2-ST and MVA-SARS-2-ST/N groups (p = 0.019 and 0.014, respectively; **Figure 3B**).

#### 3.2.2 Eyes

No morphological lesions were observed in the globes in any of the groups. SARS-CoV-2-NP-positive neurons were present in the retinas of control animals from the PBS (*n* = 2/4) and MVA-WT (*n* = 3/4) group as well as animals vaccinated with MVA-SARS-2-N (*n* = 2/8). The positive cells were mostly present in the ganglion cell layer, with one animal from the PBS and two animals from the MVA-WT group showing fewer neurons in the inner nuclear cell layer as well. No positive neurons were seen in the retinas of animals immunized with MVA-SARS-2-ST (*n* = 8) and MVA-SARS-2-ST/N (*n* = 8; **Figure 4**).

### 3.3 Emergency experiment

#### 3.3.1 Brains

All SARS-CoV-2-infected animals from both control groups (PBS and MVA-WT) developed lymphohistiocytic meningoencephalitis, a multifocal to coalescing or diffuse distribution of SARS-CoV-2-NP-positive neurons, a multifocal to diffuse presence of spiky microglia, and a predominantly perivascular T cell infiltration (**Figures 2a, b**). MVA-SARS-2-ST/N vaccinated animals showed less pronounced perivascular inflammatory changes and mostly multifocal or diffuse changes in microglia morphology. Interestingly, the T cell infiltration was not predominantly perivascular as in control groups, but also parenchymatous. Four mice from this group presented only multifocal distribution of SARS-CoV-2-NP-positive neurons with one animal showing a diffuse pattern (*n* = 4/6; **Figure 2e**). One MVA-SARS-2-ST/N vaccinated animal did not show any evidence of viral neuroinvasion or associated inflammatory changes (*n* = 1/6).

Animals vaccinated with MVA-SARS-2-ST/N exhibited a significantly lower vascular score in comparison to the MVA-WT group (p = 0.047), as well as a significant difference in the semi-quantitative SARS-CoV-2-NP score compared to the PBS (p= 0.004) and MVA-WT (p = 0.017) groups. No significant differences in the Iba1-positive area % or number of CD3-positive cells were observed in this experiment (**Figure 3c**).

#### 3.3.2 Eyes

No morphological changes were seen in the globes in any of the animals. SARS-CoV-2-NP-positive neurons were present in the retinas of three animals from the PBS group (*n* = 3/7) and in four animals from the MVA-WT group (*n* = 4/7). One animal vaccinated with MVA-SARS-2-ST/N showed viral-antigen-positive neurons in the retina (*n* = 1/7; **Figure 4**). The positive cells were observed mostly in the retinal ganglion cell layer, but two animals from the PBS, one animal from the MWA-WT and one animal from MVA-SARS-2-ST/N showed positive cells also in the inner nuclear layer.

## 4 Discussion

This study shows that double vaccination with MVA-SARS-2-S, -ST or -ST/N, as well as a single dose of MVA-SARS-2-ST or -ST/N four weeks prior to infection (Booster and Prime experiment) resulted in substantially reduced inflammatory lesions and T cell infiltration, as well as smaller proportions of Iba-1-positive areas in the brains of SARS-CoV-2-infected K18-hACE2 mice. As previously reported, MVA-SARS-2-S and -ST groups from the Booster experiment as well as MVA-SARS-2-ST/N group from the Prime study showed no clinical symptoms, no infectious virus and lower levels of SARS-CoV-2 RNA in the lungs and brains (27, 30). In the Booster study, however, animals vaccinated with MVA-SARS-2-ST showed only mild pulmonary lesions, whereas animals vaccinated with MVA-SARS-2-S presented pronounced lung pathology and additionally lower titers of neutralizing antibodies (27).

A similar vaccination regimen with intramuscular administration of one or two doses of MVA-SARS-2-S at weeks 0 and 4 followed by intranasal SARS-CoV-2 challenge at week 9 has been tested in K18-hACE2 mice by other research groups (22, 24, 25). In these studies, animals vaccinated twice were protected from weight loss and lethal outcome, had no infectious virus as well as a lower viral RNA load in the lungs. Mice immunized only once initially lost weight but eventually recovered and survived. However, they presented more prominent lung lesions than controls and had high levels of viral RNA and infectious virus (24). Moreover, both once and twice immunized mice showed no viral antigen, no viral RNA and no infection-associated lesions, including neuronal loss, vascular changes and marked microgliosis in the brain (25). Although the vaccination protocol employed in the study presented in this manuscript involved shorter intervals between vaccine administration (weeks 0 and 3) and subsequent infection only four weeks later, it was shown that neuroprotection induced by 2-time MVA-SARS-2-S vaccination also included a substantial decrease in the SARS-CoV-2 associated inflammatory lesions in K18-hACE2 mice which was consistent with previously published studies evaluating the CNS (25).

In another study, a MVA vaccine expressing a full-length prefusion-stabilized SARS-CoV-2 S protein, called MVA-CoV2-S(3P), was investigated in K18-hACE2 mice (28). The animals subjected to a single vaccination 4 weeks prior to SARS-CoV-2 infection showed full protection in terms of body weight loss, lethality, pulmonary pathology, and viral invasion, which corresponds with the findings of the Prime study showing almost no brain pathology. This suggests that clinical symptoms and lethality in this animal model may be caused by brain rather than lung pathology.

A different approach with MVA expressing explicitly a trimeric receptor binding domain (RBD) of the S protein (part of S1 domain) resulted in the survival of only half of the SARS-CoV-2 challenged K18-hACE2 mice when tested as homologous regimen (33). Although the receptor binding domain is the main target of neutralizing antibodies, other mechanisms such as cellular immunity and non-neutralizing antibodies, which are predominantly elicited by regions outside the RBD, seem to be crucial to induce full protection in this animal model. Another group utilizing wild-type mice demonstrated that MVA-based vaccine expressing a trimeric stabilized S protein induced a stronger immune response and provided better protection against infection with a mouse adapted SARS-CoV-2 strain, than a comparable vaccine expressing the antigen in monomeric form (34). These findings highlight that not only the type of antigen, but also its structural form, plays a critical role in vaccine efficacy.

This study is the first to show a successful reduction of SARS-CoV-2 induced CNS pathology in K18-hACE2 mice vaccinated once or twice with a multivalent MVA-based vaccine expressing the N protein in addition to the ST protein. Similarly, Dangi, Class (35) demonstrated that a single immunization with an adenovirus vector expressing both S and N proteins significantly reduced the viral RNA load in the brains of SARS-CoV-2-infected K18-hACE2 mice. The use of multivalent vaccines with both S and N proteins has been shown in various animal models to provide protection against SARS-CoV-2 infection, including different variants of concern (20, 21, 35–37). Although previous studies have shown that double or single vaccination with MVA-SARS-2-ST/N stimulated higher titers of neutralizing antibodies than the MVA-SARS-2-ST (27, 30), in the present study both vaccination regimens minimalized the CNS pathology to a similar extent.

In general, it has been shown in different animal species that intramuscular administration of MVA leads primary to the infection of the major histocompatibility complex class II (MHC II) positive antigen presenting cells which can display the antigen expressed by the MVA vector in the context of both MHC class I and class II molecules, leading to activation of CD8^+^ and CD4^+^ T cells respectively (38, 39). MVA-based vaccines expressing SARS-CoV-2 S protein, which protect K18-hACE2 mice from the severe course of SARS-CoV-2 infection stimulate not only the production of virus-neutralizing and non-neutralizing antibodies before and after SARS-CoV-2 challenge, but also induce S-specific CD4^+^ Th1-biased responses as well as CD8^+^ T-cell–mediated cellular immunity (22, 24, 27, 30). Reduced production of pro-inflammatory cytokines including IL-6, TNF-α and IFN-γ within the lung and brain tissue upon SARS-CoV-2 infection has also been reported (24, 40). However, the magnitude and quality of the induced immune responses vary depending on the specific design of the vaccine, as highlighted above.

While discussing the possible mechanism of neuroprotection in K18-hACE2 mice it is important to understand that the pattern of widespread viral dissemination throughout the CNS does not appear to recapitulate the typical course of SARS-CoV-2 infection in humans. In human patients, evidence of neuroinvasion remains limited and controversial (10–12, 41, 42). SARS-CoV-2 RNA or protein has been detected in the brains of patients with COVID-19 less frequently and at substantially lower levels than those reported in this animal model. Therefore, findings obtained from the transgenic K18-hACE2 mice should be interpreted with caution in terms of their translational relevance. The distinct course and characteristics of infection, particularly the pronounced neuroinvasion, suggest that some of the underlying pathogenic mechanisms may be specific to this model. Indeed, other animal models, such as wild-type mice and golden Syrian hamsters, do not exhibit similarly extensive CNS involvement.

The data obtained within experiments presented in the previous (27, 30) as well as in this publication show that the vaccinated and infected animals in which no infectious virus and viral protein was detected within the brain, show none or only minimal inflammatory changes. This implies that lower viral burden within the CNS leads to reduction of the local inflammation. Naive T cells are primed and become antigen-specific in the lymph nodes that drain initial sites of viral replication, rather than within the CNS (43). Since the CNS represents a unique immune environment, only a small number of T cells cross the blood–brain barrier (BBB) under homeostatic conditions. However, during peripheral infection, pro-inflammatory cytokines alter BBB permeability and increase the expression of adhesion molecules, facilitating enhanced T cell trafficking into the CNS. If infiltrating T cells do not encounter the specific antigen for which they were primed, they either undergo apoptosis or re-enter the circulation. In contrast, when their specific antigen is present within the CNS, T cells are retained in the perivascular spaces and can subsequently migrate into the parenchyma to exert effector functions (43). It remains unclear whether the limited initial replication of the virus within the respiratory tract (24, 30) and high titers of neutralizing antibodies suffice to completely prevent viral entry into the CNS or whether the vaccine-induced immune response instead reduces the ability of neurons to support viral replication which enables the clearance of the virus that had entered the CNS. It also remains to be fully elucidated whether humoral or cellular immune response is essential for the neuroprotection in this animal model. Nevertheless, some findings indicate that neutralizing and binding antibody titters correlate with protection against severe infection in this model (24). This is further supported by a study conducted in K18-hACE2 mice vaccinated with mRNA vaccine and subsequently challenged with SARS-CoV-2 Alpha strain in which only vaccinated K18-hACE2 mice with B cells were protected from severe infection course and showed substantially lower viral RNA levels in the brain whereas only 20% of the vaccinated B cell deficient K18-hACE2 mice survived the infection and there was no difference in CNS viral RNA loads in the vaccinated and unvaccinated animals, suggesting that humoral response might be a substantial part of vaccine-induced neuroprotection in this animal model (44).

In contrast, no neuroprotection was observed in animals immunized with vaccines expressing only the N protein. These findings coincide with a previous study conducted with Ad5-hACE2 (adenovirus expressing hACE2) transduced BALB/c mice, in which the administration of anti-N immune serum did not protect the animals from subsequent SARS-CoV-2 infection (45). Although N protein is highly immunogenic and induces mainly cellular, but also humoral immunity, N-specific antibodies lack neutralizing activity as described by others (46, 47). This could explain the lack of protection from acute SARS-CoV-2 infection. However, it would be interesting to evaluate the long-term effects of heterogenous immunization regimens or multivalent vaccines that would also include the N protein.

In the Emergency study, the inflammatory lesions induced by a SARS-CoV-2 challenge infection only 2 days after a single MVA-SARS-2-ST/N vaccination were investigated. Milder perivascular inflammatory brain lesions were observed in comparison to the control groups. In addition, there was a slight increase in the numbers of T cells, which interestingly were not only located in the perivascular compartment, but also in the brain parenchyma. As previously published, the vaccinated animals from this experiment showed milder clinical symptoms and survived longer than control animals. Furthermore, they presented a lower titer of infectious virus as well as viral RNA load in the lungs, but not in the brain (30). However, immunohistology for viral N protein revealed a lower number of positive cells in the lungs and a lower positive area percentage in the brain of vaccinated animals compared to controls (30), which corresponds with milder inflammatory changes in the brains reported in this study. These differences could possibly be attributed to the more prominent humoral and cellular immune response in vaccinated animals (20–22, 24, 30).

Of note, MVA-based vaccine candidates encoding the native and prefusion-stabilized SARS-CoV-2 spike protein proceeded to phase one clinical trials (20, 27, 48, 49).

It is important to point out that the timing of animal sacrifice, which ranged from 6 to 8 dpi should be acknowledged as a limitation of these experiments, as it may have influenced the determined degree of severity of the observed pathological changes. However, most studies evaluating the spatiotemporal distribution of CNS lesions in infected K18-hACE2 mice have shown that inflammatory changes and neuronal viral antigen loads remain present at later stages of the infection and have not reported significant differences between these timepoints (25, 31). Additionally, CD3 and Iba-1 markers utilized in this study allow broad assessment of overall T cell and microglial/macrophage infiltration and morphology, however, to further characterize these cell populations additional investigation using FACS and single cell RNA-seq analysis would be required.

In conclusion, K18-hACE2 mice immunized once or twice four weeks prior to infection with vaccines containing SARS-CoV-2 S protein (native or stabilized) were protected from the viral spread to the brain and retina and showed none to minimal inflammatory changes in the brains. Animals vaccinated with MVA-SARS-2-ST/N two days prior to infection showed fewer viral antigen positive neurons in the brain and retina as well as milder inflammatory lesions in the brain compared to controls. However, further studies, possibly with extended timelines would be required to evaluate if this observation is associated with delayed or permanently milder disease course.

## Data Availability

The original contributions presented in the study are included in the article/supplementary material. Further inquiries can be directed to the corresponding author.
